# Abdominal complications of ascariasis in childhood

**DOI:** 10.1016/j.jped.2024.02.001

**Published:** 2024-03-21

**Authors:** Ivonete Siviero, Ugo V.B. de Almeida, Claudia R.R. Penna, Elazir B.M. Di Puglia, Betina C. Bertrand Simões

**Affiliations:** a Universidade Federal do Rio de Janeiro (UFRJ), Faculdade de Medicina, Departamento de Cirurgia, Rio de Janeiro, RJ, Brazil; b Universidade Federal do Rio de Janeiro (UFRJ), Instituto de Puericultura e Pediatria Martagão Gesteira (IPPMG), Rio de Janeiro, RJ, Brazil

**Keywords:** Ascariasis, Surgical complications, Child, Intestinal obstruction, *Ascaris lumbricoides*

## Abstract

**Objective:**

Complications of ascariasis are a significant cause of abdominal pain in pediatric emergencies, especially where it is endemic. A literature review was conducted with the aim of improving diagnostic and therapeutic approaches for these patients.

**Data sources:**

A PubMed search was conducted using the key terms “ascariasis complications” and “hepatobiliary ascariasis”. The search strategy included meta-analyses, randomized controlled trials, clinical trials, observational studies, case reports, and reviews published up to December 2023.

**Summary of findings:**

Obstruction of the small bowel is the most common complication. Others that are, rarer and more difficult to properly identify and treat, such as biliary, hepatic, and pancreatic complications, acute appendicitis, Meckel's diverticulitis, or peritoneal granulomas. Hepatic and pancreatic complications are rarer and more serious in children than in adults. While plain radiography is usually the only option in cases of intestinal obstruction, ultrasonography is the examination of choice in cases of hepatobiliary, pancreatic, and appendicular ascariasis complications in childhood. The treatment is clinical and conservative in most patients. Surgical treatment is indicated if conservative therapy fails, or if there are signs of complications. Laparoscopy has been used as an excellent technical alternative in adults with hepatobiliary complications of ascariasis, but further studies on its use in children are still needed.

**Conclusion:**

The creation of protocols and greater debate on this subject should be encouraged for a better understanding of the disease and to establish an early diagnosis and adequate treatment for children with complications resulting from massive infestation by *Ascaris lumbricoides*.

## Introduction

Ascariasis is a helminthic infestation caused by *Ascaris lumbricoides* (*A. lumbricoides*), with a global distribution, affecting approximately one billion people, or about 24 % of the world population.^1^ It occurs mainly in tropical and subtropical areas, with the largest numbers recorded in Africa, the Americas, China, and East Asia.^1^ The disease is associated with poor hygiene, lack of access to water, and inadequate sanitation. It is classified as a neglected disease by the World Health Organization (WHO).^1^

Ascariasis is transmitted through the fecal–oral route. Infection occurs when embryonated eggs that contaminate food, utensils, or hands are ingested. The eggs hatch in the small intestine, releasing the larvae that pass through the intestinal wall and migrate through the liver and heart, up to the lungs. In the lung passage, the larvae are expectorated and swallowed, passing through the gastrointestinal tract until they arrive at the small intestine, where they mature into adult worms and produce new eggs which are expelled with feces contaminating the environment. Reinfection occurs only when contaminated eggs are ingested. The adult female worm produces around 240,000 eggs/day. Humans are the reservoir and the only definitive host of Ascaris lumbricoides.^2^

The prevalence and intensity of *A. lumbricoides* infestation commonly reach the highest levels among children aged 5–10 years.^2^ Although the disease can occur at all ages, it is rarer before 2 years of age and after 15 years of age.^3^^,^^4^ It is estimated that this disease, which can greatly impact the health and quality of life of millions of Brazilians, can cause harmful conditions such as impaired cognitive and physical development, malnutrition, and diver's symptoms such as diarrhea, abdominal pain, inappetence, or complications.^5^ In areas where ascariasis is prevalent, abdominal complications occur in a significant number of children and may correspond to up to 20 % of acute abdomen.^2^

Most *A. lumbricoides* infestations are asymptomatic or present with mild symptoms such as intermittent abdominal pain; however, severe life-threatening complications can occur. In children with high parasite loads, complications occur mainly in the small intestine, the natural habitat of the worm in humans, which causes obstructive conditions. More rarely, worms migrate to the appendix, Meckel's diverticulum, or bile ducts. In biliary ascariasis, worms may cause mechanical obstruction and inflammatory reactions of the biliary tract, resulting in biliary colic, acute cholangitis, cholecystitis, and pancreatitis. In addition, they may migrate carrying intestinal bacteria, which can cause infectious complications, such as liver abscesses and sepsis.^2^^,^^4^^,^^5^

The diagnostic investigation of the obstructive bowel condition was performed using plain abdominal radiography, where the characteristic images of the massive infestation can be identified. An abdominal ultrasound (US) may also reveal the "masses" formed by the entangled worms, or even other complications of ascariasis, such as obstruction of the appendicular lumen (acute appendicitis), biliary obstruction (cholangitis), or pancreatic obstruction (pancreatitis). Acute appendicitis is the most common differential diagnosis. Blood counts usually show anemia and leukocytosis.^2^^,^^6^ A high index of suspicion is necessary to determine the ectopic presence of the worm; however, this diagnosis should always be considered in areas where ascariasis is endemic, in children with abdominal pain, low socioeconomic status, and poor sanitation conditions.

This paper is an update on the treatment currently recommended for cases of abdominal complications caused by massive infestation of *A. lumbricoides* in childhood, with the aim of warning about this problem in pediatric emergency units.

### Intestinal complications of ascariasis

The most common complication of ascariasis is intestinal obstruction, and the most common mechanism is mechanical obstruction due to occlusion of the intestinal lumen resulting from the entanglement of many worms, mainly in the distal ileum. Although the domain of these parasites extends from the stomach to the ileocecal valve, the vast majority inhabit the jejunum and the proximal ileum. Intestinal obstruction may also occur due to intestinal volvulus or intussusception and may cause gangrene in the small intestine.^6^

In addition to the mechanical factor itself, intestinal obstruction may be due to neurotoxins excreted by the Ascaris that die during their evolutionary cycle. These proteinaceous substances cause spasms in the muscles of the small intestine, mainly at the level of the distal ileum, facilitating the formation of intraluminal masses of Ascaris and obstruction. Other toxins, such as anaphylatoxins, hemolysins, and endocrinolysins, can also cause an inflammatory reaction in the intestinal segments affected by worms. These factors make the small intestine the most common site of surgical complications of ascariasis. There are no described complications related to this parasitosis in the colon.^7^ Clinical intestinal disease depends on the parasite load. If the parasite load is small, the child is usually asymptomatic or has mild symptoms such as nausea, abdominal discomfort, and anemia. Complications occur when many Ascaris are present in the intestinal lumen.^4^^,^^7^

Patients with massive infestation may present with acute intestinal obstruction, a history of colicky abdominal pain, nausea, and vomiting, in addition to dehydration, pallor, growth retardation, and weight loss. Some children present with a slight increase in axillary temperature and pneumonitis (Loeffler's syndrome). There may be a history of elimination of the worm in vomit or feces and alterations in the intestinal habits, even with cessation of evacuation. Abdominal examination revealed distension, localized or diffuse tenderness, rigidity, palpable worm masses, or visible peristalsis may be noted.^4^^,^^6^ The characteristic Ascaris bolus or palpable masses, which correspond to the worms coiled together and their frequent change of location in subsequent abdominal examinations, is one of the peculiarities of this disease.^2^

As for the distribution of pain, the periumbilical region is the most common since the jejunum and proximal ileum are the most common natural habitats of A. lumbricoides. Abdominal distension usually occurs later and may be due to associated metabolic ileus6. In the periumbilical region, it is possible to palpate worm masses most of the time. An Ascaris bolus of approximately 5 cm was the most easily palpable. As already mentioned, its peculiarity is the frequent change in position during subsequent abdominal examinations.^6^ If the condition develops into complications such as intestinal gangrene and peritonitis, in addition to abdominal pain and distension, the child may present with increased temperature and dehydration.^2^^,^^6^

Intestinal intussusception caused by ascaris is rare and may include the ileal jejunum, ileal ileum or jejunal jejunum. Intestinal volvulus may cause ischemic necrosis and intestinal perforation if it is not diagnosed and treated quickly. In these cases, besides the suggestive picture of intestinal obstruction, the child usually presents signs of toxemia.^7^

The cause of perforation of the small intestine remains controversial, with two main theories. During extreme conditions, such as inflammation, starvation, or worm bolus obstruction, some parasites are believed to migrate into the ulcers and cause perforations. Another possible explanation is that the large worm bolus can lead to pressure necrosis and gangrene. The intestine has immense capacity to expand, and it has been claimed that it can accommodate 5.000 worms without symptoms occurring. It is thus unlikely that direct pressure by ascarids can produce intestinal perforation.^8^

Ascariasis in Meckel's diverticulum may cause diverticulitis, perforation, or gangrene9. Although *A. lumbricoides* is known to be present in the lumen of the vermiform appendix, it is rare, and its presence is rarely associated with appendicitis.^9^

The identification of Ascaris eggs in stool samples is diagnostic. Blood counts usually show anemia and leukocytosis. Eosinophilia, which is present in the early stages of infestation, is not diagnostic and is usually absent in the setting of acute intestinal obstruction or other complications of ascariasis. The diagnosis of intestinal complications of ascariasis was based on data from history, physical examination, and radiological findings.^1^

**Figure 1:**
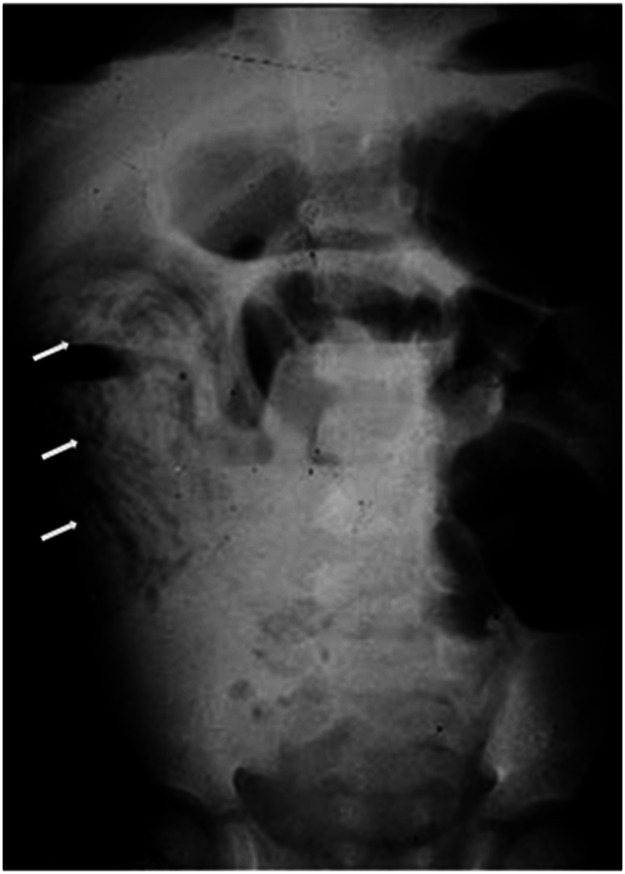
Abdominal plain abdominal X-ray showing intestinal obstruction by Ascaris (arrows).

Plain abdominal radiography revealed signs of hydro-air levels and dilatation, major or minor, of the loops in their presence of bowel obstruction or sub-occlusion, in addition to images of *A. lumbricoides*. The worms are seen as longitudinal images or in cross-sections, described as a "swirl”, "breadcrumb" or "cigarette pack" pattern, which corresponds to the parasites tangled in the intestinal lumen (Figure 1). These findings, are associated with history and physical examination, are sufficient to establish a diagnosis.^4^^,^^6^, ^7^, ^8^ In addition, plain radiography is important for the serial assessment of the progress of conservative treatment.

**Figure 2:**
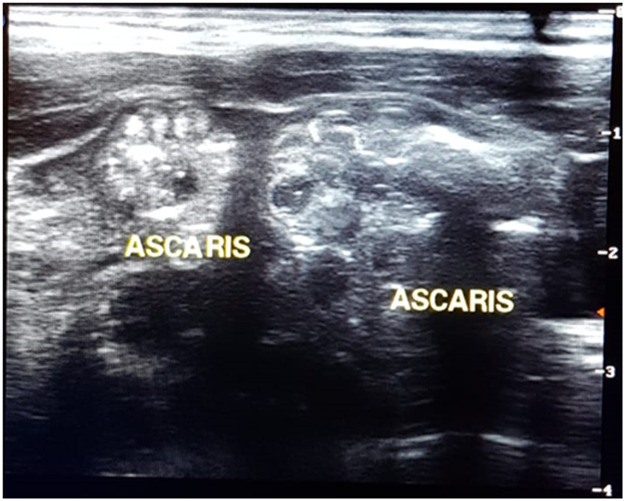
Abdominal ultrasound showing *A. lumbricoides* in the intestinal lumen.

The abdominal US may also highlight the presence of worms within the intestinal lumen, a dilated intestinal loop with a thickened wall, and a mass of worms causing obstruction. Common sonographic features are usually described as thick, echogenic linear bands with central anechoic tubes and no acoustic shadowing, or looking like a "winding road,” "parallel lines,” "railway sign,” "inner tube sign" or "target",^9^, ^10^, ^11^ as shown in Figure 2. The US has a sensitivity of up to almost 80 % and may also be useful in highlighting other complications of ascariasis, such as obstruction of the appendicular lumen causing acute appendicitis and biliary or pancreatic complications.^4^

Contrast-enhanced computed tomography or magnetic resonance imaging may be useful, but are not essential, especially in acute cases, and may be dispensed. In most cases, a simple abdominal radiography is the only necessary examination. The investigation may be performed or complemented, in cases of complications in US.^2^^,^^7^ The main differential diagnosis is acute appendicitis because of the similarity of signs and symptoms between the two diseases.^7^

Uncomplicated ascariasis can be successfully managed with conservative management, with zero oral diet, nasogastric tube (NGT) suction, hydro electrolytic resuscitation, and an enema of 0.9 % or hypertonic saline solution, with a therapeutic success of 50–85 % of cases.^6^^,^^9^^,^^12^ Therefore, if there are no signs of complications such as peritonitis or pneumoperitoneum and intestinal gangrene, conservative management is indicated in cases of obstruction or sub-occlusion by ascaris.

Gastrografin has also been used successfully in the conservative management of patients with sub-occlusion and/or intestinal obstruction by Ascaris without complications (volvulus and gangrene).^4^ It is a hyperosmolar agent administered via the NGT, which promotes the displacement of fluid into the intestinal lumen and increases the pressure gradient at the site of obstruction. It also promotes a decrease in edema of the intestinal wall and increases intestinal motility. With excess fluid in the intestinal lumen, the worms separated, allowing their elimination. It also acts as a wetting agent, causing the worms to slip and pass into the colon to be eliminated within 1–3 days. Gastrografin is radiopaque, enabling radiological and clinical follow-up of the evolution of the condition. A study by Hamid et al demonstrated that the use of gastrografin resulted in faster relief of signs and symptoms of Ascaris lumbricoides-induced intestinal obstruction, the earlier passage of worms and flatus, and faster return to oral feeding, and that its adverse effects were less than those of hypertonic saline enema.^11^ Patients undergoing treatment with gastrografin should strictly control their hydration to avoid clinical complications such as dehydration.

With conservative treatment, the cakes become undone and smaller, allowing their passage through the ileocecal valve. The time interval for the disappearance of Ascaris cakes is generally 24 to 96 h with conservative management.^6^ The criteria for evaluating the success of conservative treatment are the disappearance of colicky pain, the beginning of defecation, or the disappearance of hydro-air levels or palpable masses.^6^^,^^13^

After resolution of sub occlusion or uncomplicated obstruction with conservative treatment, the use of anthelmintics, such as albendazole (400 mg single dose from 2 years old) or mebendazole (100 mg kg^-1^ twice a day for 3 days for patients older than 12 months) should be initiated and repeated 6 weeks after discharge for eradication of the worm.(4,6) In children under 2 years of age a single dose of 200 mg of albendazole on one day produces good efficacy. In children aged 12 to 24 months, albendazole can be used at a dose of 200 mg/dose, after assessing the risk/benefit of its use, as there are no studies guaranteeing its use in this age group. Should not be used in children with encephalopathies or hepatopathies, as it may aggravate these conditions. Albendazole may be slightly more effective than mebendazole, but both have a rate of reduction in stool egg counts of over 95 % after a single dose. Both albendazole and mebendazole can cause transient abdominal pain, nausea, or diarrhea in children infected with many worms.^4^^,^^6^

Studies have highlighted that the administration of anthelmintics in children with abdominal pain and obstruction may worsen the clinical picture and lead to serious complications such as intussusception, volvulus, intestinal bleeding, necrosis, and perforation. This is because these medications can cause complete paralysis in many worms, which would accumulate in the distal small intestine, blocking its lumen.^4^

In some centers worldwide, another alternative in the conservative management of subocclusion or even intestinal obstruction is the use of mineral oil via NGT until its elimination via the rectal route. The oil helps in the elimination of the worm via the rectum and certifies that there is continuity of intestinal transit. With the patient under venous hydration, disinfestation with piperazine was performed after elimination of the oil, disinfestation with piperazine is performed.^4^^,^^7^ This is abolished in developed countries owing to the toxic effects of piperazine.^11^ In Brazil, their use has been suspended since 2008.^14^ The proscription of mineral oil, which has been widely used in various therapies, was mainly due to its risk of causing lipoid pneumonia (LP), a much-feared complication that is difficult to diagnose. In children, the most common LP is exogenous owing to the aspiration of mineral oil for use in constipation or subocclusion by Ascaris. Its high viscosity may reduce cough or choking reflex, facilitating aspiration, even in the absence of risk factors. In addition, it may carry germs to the lower airway, which is responsible for recurrent acute respiratory infections.^15^

Distinguishing between simple bowel obstruction and intestinal distress due to ischemia can be difficult, making regular critical assessment necessary until normal bowel function has returned. Urgent clinical resuscitation and laparotomy are required for a very sick and toxic child with a distended, tense abdomen, with signs of peritoneal irritation, and in the presence of complete bowel obstruction or unresolved by conservative treatment or with pneumoperitoneum. Elimination of blood through the rectum is a sign of severity.^2^

The following are the criteria for indication of surgical treatment: 1) unsatisfactory response to conservative management; 2) toxemia disproportionate to the severity of the intestinal obstruction; 3) increased abdominal distention, tenderness, or painful decompression; 4) persistent abdominal pain and tenderness at the site of the worm mass; 5) persistence of the worm mass at the same site on abdominal examination; 6) rectal bleeding; 7) increased distension of intestinal loops and number of hydro-air levels; 8) any evidence of volvulus or intussusception; 9) presence of pneumoperitoneum; 10) sonographic evidence of significant and/or progressively increasing increase in worm cakes, free fluid in the peritoneal cavity, and any evidence of peritonitis.^6^

Toxic patients require aggressive management with intravenous infusion of crystalloids, such as ringer lactate solution or adrenergic agents, and broad-spectrum antibiotic therapy. Only after stabilization should the patient undergo surgical treatment with exploratory laparotomy. When viable intestines are found, an enterotomy can be performed to extract the worms. Milking of worms should be avoided as this may cause lacerations of the serosa on the intestinal wall or rupture of the worms, causing the release of their toxins. If intestinal compromise is present, intestinal resection and end-to-end anastomosis should be performed. Temporary stomas are rarely needed.^4^^,^^16^ In cases of intestinal volvulus, distortion, and enterotomy at the point of maximum worm burden should be performed to extract the worms, provided that the intestinal wall.(9) Postoperative complications include wall abscesses, sepsis, anastomotic dehiscence, and respiratory infections. Mortality ranged from 1 to 8 %.^6^

Early diagnosis, surgical intervention as soon as possible, when necessary, coverage with adequate antibiotic therapy and radiological evaluation are factors that reduce the morbidity and mortality of children with intestinal obstruction by *A. lumbricoides*.^6^

The WHO recommends periodic disinfestation, even without a prior individual diagnosis, for all people living in endemic areas.^1^ This intervention reduces the worm burden and, morbidity. In addition, health and hygiene education encourages healthy behavior and reduces transmission and reinfection. The provision of adequate sanitation is also an important but not always possible, measure in resource-poor settings. Treatment and prevention programs must be strictly followed to reduce the number of intestinal complications associated with this worm. Intestinal obstruction due to *A. lumbricoides* should always be a diagnostic hypothesis in cases of acute abdominal pain in endemic areas.

### Biliary, hepatic, and pancreatic complications

Complications of ascariasis in the hepatobiliary system are less frequent in children (5 %) than in adults (53 %), because of the small ductal caliber in childhood. They occur more frequently in the endemic areas of India and South Africa.^17^, ^18^, ^19^

*A. lumbricoides* tend to enter all orifices and carry the intestinal flora. If there is a massive worm burden in the duodenum, there is a greater likelihood of biliary ascariasis. Along with worm migration, bacterial migration can lead to inflammation and infection. In biliary ascariasis, one or two worms usually enter the biliary system, although there may be many of them. The irritation caused by the worm, or the result of its excretion causes biliary colic and spasm of the sphincter of Oddi with partial biliary obstruction. Both secretions from the worms and their breakdown in the extrahepatic biliary system can lead to an intense inflammatory response, resulting in cholangitis, stenosis, fibrosis, lithiasis, calcification, or ductal necrosis.^18^^,^^19^

The presence of Ascaris in the biliary tree may cause acute conditions such as biliary colic, or more severe conditions such as acute cholecystitis, acute cholangitis, acute pancreatitis, and even liver abscess.^10^

Hepatobiliary and pancreatic complications of ascariasis should be suspected when a child infested with this worm presents with acute onset abdominal pain, which can be colic-like, especially in the right upper quadrant, vomiting, mucocutaneous pallor, and elimination of worms in vomit or feces. It usually has a low fever, but in the presence of pancreatitis or liver abscess, there may be a higher fever, in addition to painful decompression, hepatomegaly, and changes in liver function.^18^

Acute cholangitis is an emergency presenting with high fever, chills, jaundice, and upper abdominal pain. The patient presented with hypotension, painful hepatomegaly, leukocytosis, elevated bilirubin (mainly conjugated), and increased liver enzyme levels, mainly glutamic-pyruvic transaminase and alkaline phosphatase.^17^

Pancreatitis associated with ascariasis usually occurs because of obstruction of the common bile duct and may evolve with the formation of pancreatic pseudocysts. In endemic regions, up to 20 % of all pancreatic pseudocysts may be caused by pancreatic ascariasis. Invasion of the pancreatic duct is rare in childhood because of its small caliber.^15^ Pancreatitis caused by ascariasis can be severe or even fatal.^10^

Although rare, a liver abscess can develop, because of bacterial migration with the worm, mainly *Escherichia coli*. They are usually more severe in childhood than in adulthood. It may be solitary or multiple and contains pus. There was painful hepatomegaly, high fever, tenderness, and pain in the right hypochondrium. These abscesses may result from dead eggs released by female worms migrating into the common bile duct, producing a granulomatous inflammatory reaction with subsequent collapse with infiltration of eosinophils or by invasion of the intrahepatic ductal system by the worm.^17^^,^^20^ Pus aspiration of liver abscesses may reveal Ascaris eggs.^17^

The diagnosis of hepatobiliary and pancreatic complications of ascariasis is difficult. It is a diagnosis that must be considered in areas endemic to the disease and a suggestive clinical picture. When this is suspected, the US is the most useful diagnostic method because it is highly sensitive and specific for visualization of the worm in the biliary system, allows monitoring of ductal mobility over time, and is fast, safe, and non-invasive. It determines the anatomical position of the worms, their motility and number, the status of the intra and extrahepatic biliary system, and possible intrahepatic and pancreatic complications. When visualized along the longest axis, the worms appeared as linear echogenic structures without acoustic shadowing. Their motility can be observed in real-time in the biliary tract or intestine.^10^^,^^17^, ^18^, ^19^

**Figure 3:**
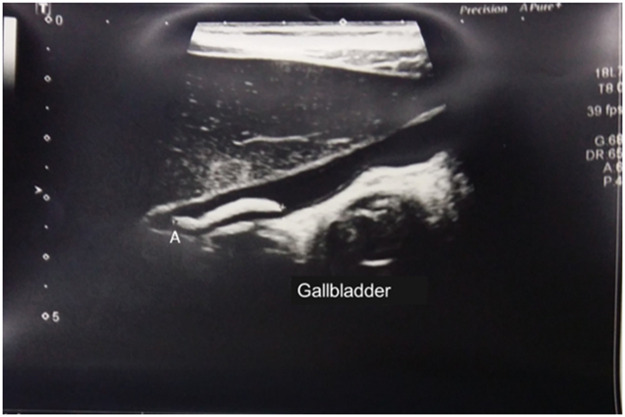
Biliary tract ultrasound. Note Ascaris in the gallbladder (A).

In the patient with ascariasis in the biliary tract and US may have any of the following imaging features: "inner tube sign" in which the worm can be seen as a thick echoic band with a central longitudinal anechoic tube (gastrointestinal tract of the worm) in the gallbladder or common bile duct; "spaghetti sign,” with overlapping longitudinal interfaces in the main bile duct; linear calcified structures within the bile ducts that may correspond to dead and calcified worms, representing old hepatobiliary infestation; "target sign,” when parasites are visualized in a dilated bile duct.^10^^,^^18^^,^^19^ Worms may also be seen protruding into Vater's papilla. (20) Calcified worms in the intrahepatic duct are occasionally seen on routine US in asymptomatic patients in regions where the disease is endemic.^10^ The presence of the worm within the gallbladder is very rare but is easily detected in the US, as illustrated in Figure 3.^10^ In cases of acute cholecystitis, it is more common to identify a distended gallbladder with a biliary sludge.^17^

Ultrasonography (US) is an excellent method for diagnosing liver abscesses due to ascariasis. It was seen as a hyperechoic area with irregular margins. The presence of worms in the duodenum supports this diagnosis.^19^

Magnetic resonance cholangiopancreatography can also be used in the diagnosis of hepatobiliary ascariasis, but it is an expensive and often inaccessible exam, making it unnecessary, as it can be performed using typical images seen on US.^10^

CT scans reveal the worms as cylindrical structures and can be used for better visualization of the dilated ductal system; however, its use should be very judicious and restricted in childhood due to the excessive exposure to ionizing radiation to which the child will be subjected.^17^

Endoscopic retrograde cholangiopancreatography (ERCP) plays an important role both in the diagnosis of *A. lumbricoides* infestation in the biliary and pancreatic ducts (where serpiginous images may be observed, causing filling defects) and in the treatment of this disease by, removing the worms and allowing decompression of the bile duct, with or without sphincterotomy.^17^^,^^18^^,^^21^

More than 95 % of patients with uncomplicated biliary ascariasis respond to conservative treatment, and worms in the bile ducts spontaneously return to the intestine. Conservative therapy can be successful even when there is massive infestation of the bile ducts if there is no invasion of the liver.^19^ Conservative treatment consists of hydro electrolyte replacement, broad-spectrum antibiotic therapy, and antispasmodics for at least 72 h.^17^^,^^18^ In cases of acute pyogenic cholangitis, more specific antibiotics are indicated, depending on the biliary pus culture and sensitivity results.

Oral anthelmintics paralyze the adult worm and should only be administered if the patient passes gas or feces. With clinical treatment, the worm is usually eliminated within 1–3 days in most cases.^17^ It is possible to wait several days or 2–3 weeks for worm excretion using anthelmintics, provided the clinical picture does not show complications.^17^

Endoscopic removal (ERCP) of bile duct worms favorably affects outcomes and is an effective alternative to surgery.^18^ When complications are suspected, an ERCP should be performed as soon as possible to remove worms from the common bile duct and remnants of the worm from the extrahepatic biliary system.^18^^,^^20^

Surgery is reserved for patients who do not respond to conservative therapy, those with liver invasion, or those in whom ERCP fails. Removal of the worm is possible in 55–89 % of cases. In a study by Bahu et al, endoscopic removal was possible in 4 of 7 patients (57 %). In most cases, acute pyogenic cholangitis requires decompression or biliary drainage in most cases.(15) Surgery is also indicated in cases of biliary stenosis or worms in the gallbladder, surgery is also indicated.^17^

Acute pancreatitis and, intestinal obstruction with complications such as volvulus, gangrene, or perforation may be present along with hepatobiliary ascariasis. It is difficult to diagnose and must be treated appropriately. Pancreatitis secondary to ascariasis may evolve with pseudocyst formation.^17^^,^^18^

Laparoscopic removal has been used successfully, mainly in adults, as a technical alternative in patients in whom conservative or endoscopic treatment failed to eliminate bile duct worms. Cholecystectomy is advocated because of the increased risk of lithiasis and the fact that the gallbladder represents a reservoir for parasites, choledochotomy, and exploration of the common bile duct.^20^^,^^22^, ^23^, ^24^

Although newer antimicrobials have improved the treatment of liver abscesses, surgery remains the standard treatment, particularly in cases of multiple abscesses or associated bile duct obstruction. This is particularly true in Ascaris-induced liver abscesses, as the adult worm and mixed intestinal flora invade the liver parenchyma. Large abscesses usually require percutaneous ultrasound-guided or surgical drainage. The mortality rate of ascaris liver abscesses can reach 10 %.^17^^,^^18^

The mortality rate of hepatobiliary complications is low, and the prognosis is good in most cases; however, epidemiological, and immunological aspects of infection associated with this worm disease are still not completely clear, making it difficult to define these cases in children.^17^

Relationship between ascariasis and gallstones: Any biliary obstruction, complete, incomplete, or recurrent, leads to impaired biliary drainage with secondary bacterial infection, mainly by *E. coli*, which produces beta-glucuronidase and unconjugated bilirubin glucuronides in bile. The resulting unconjugated bilirubin precipitates with calcium to form calcium bilirubinate stones. This provided a suitable environment for gallstone formation. Adult worms, eggs, and larvae can initiate gallstone formation.^17^

### Rare abdominal complications of ascariasis

Ascaris granulomatous peritonitis is a rare complication of intestinal ascariasis, which occurs due to the passage or perforation of the digestive tract by the adult worm into the peritoneal cavity, where it deposits its worms, causing an intense granulomatous inflammatory reaction.^23^, ^24^, ^25^

Other rare complications include surgery. There have been reports of gastrointestinal bleeding, intestinal perforation, biliary perforation, neobladder perforation, gastric perforation, postoperative obstruction of the nasogastric tube, Kehr drain, or jejunostomy tube obstruction.^5^^,^^8^^,^^21^^,^^26^, ^27^, ^28^

## Discussion

This literature review clarifies that abdominal complications of ascariasis go beyond intestinal obstruction and that even in these cases, there is a need to establish an early diagnosis to avoid an unfavorable outcome. Less frequent complications, which are no less important in pediatric clinical practice, involve great diagnostic and therapeutic difficulties.

There are not many large series or updated scientific studies on the subject, and those available are from countries where ascariasis is endemic, often neglected, and with very poor sanitary and public health conditions. There is a need to establish protocols, early diagnosis, and adequate therapy to avoid these complications and, above all, to establish adequate sanitary and collective health policies for these populations. In Brazil, there are still very poor regions with difficulties in these aspects, with more complications from this disease.

It is essential to know all the clinical aspects of the disease, which exams are more indicated in each child, and define which ones will benefit from conservative treatment, which is efficient in most cases if the diagnosis is early. A high degree of suspicion is necessary to avoid complications, especially rarer ones, such as hepatobiliary and pancreatic complications. This hypothesis must be confirmed or ruled out, especially if the child comes from an endemic area or from a low socioeconomic and sanitary-level area, which is common in Brazil.

In the case of a child with intestinal obstruction caused by A. lumbricoides, it is necessary to have regular, continuous, and intense follow-up to note a change that indicates the need for surgical treatment. Gastrografin has been shown to be an effective alternative for treating cases of intestinal obstruction. The use of saline enemas or gastrografin through a nasogastric tube associated with anthelmintic treatment, after the resolution of the obstructive picture, has replaced clinical management with mineral oil and piperazine, which were proscribed in Brazil.

In such cases, there is no doubt about the need for surgical treatment, such as intestinal volvulus, intussusception, or pneumoperitoneum caused by worms. There is a need for rapid clinical compensation, broad-spectrum antibiotic therapy, and immediate surgical treatment because this directly impacts the outcome of the treatment.

Hepatobiliary and pancreatic obstructions, more common in adults, present with greater severity in childhood, and treatment is more difficult and specialized, as there is less availability of appropriate equipment and greater technical difficulty in performing retrograde cholangiopancreatography, which is the treatment of choice in this age group. Gallstone formation may further aggravate these cases or lead to late complications.

While plain radiography is the diagnostic method of choice and is usually the only method in cases of intestinal obstruction, ultrasonography is the examination of choice in cases of hepatobiliary and pancreatic complications of ascariasis in childhood. The use of a CT scan, with its high ionizing radiation load, for this type of complication is an exception in children with this condition.

Laparoscopy has been used as an excellent technical alternative in adults with hepatobiliary complications of ascariasis, but further studies on its use in children are still needed.

Even rarer complications such as granulomatous peritonitis, perforation of hollow viscera, and drains or probes have been sporadically described in the literature.

The creation of protocols and further debate on this subject should be encouraged for a better understanding of the disease and to establish an early diagnosis and adequate treatment for children with complications resulting from massive infestation by A. lumbricoides.

## Conflicts of interest

The authors declare no conflicts of interest.
